# Supplementary material for: The liminal space between hope and grief: The phenomenon of uncertainty as experienced by people living with relapsing-remitting multiple sclerosis
Source: PLoS One. 2025 Jan 28;20(1):e0315501. doi: 10.1371/journal.pone.0315501 (PMC11774396; doi:10.1371/journal.pone.0315501)
Supplement: S1 Appendix — (PDF) [file pone.0315501.s001.pdf]

Appendix 1. The interview guide

1. Introduction

- Expression of gratitude for participation and time.
- Response to possible questions rising from information letter and additionally provided information about research project.
- Information about participation and further procedures: voluntary nature, pseudonymity, recording of interview, ad verbatim transcript, followed by signing of the informed consent form.
- Information about the interview itself: its open character, focus on experiences rather than opinions and deep probing into details.
- Room for further questions.

2. Interview

Introductory question: what does uncertainty pertaining to having MS mean to you?

Diagnosis
- How did you experience uncertainty during the time of receiving the diagnosis?
- Can you describe a moment back then that felt uncertain to you?

Medication
- Can you take me back to the first time the possibility of starting medication was mentioned to you? What was this conversation like?
- How did you reach a decision on starting medication and what type to use?
- What were the first few days of taking medication like? (if applicable)
- Do you experience specific uncertainties relating to using medication and if so, can you describe them to me? (if applicable)

Daily life
- Can you describe when and how you experience uncertainty in your daily life? What do the uncertainties relate to?
- How do you deal with this? What role does your doctor/family/friends play in this?
- Can you describe a moment of heightened uncertainty?
- Can you describe a moment or phase when the uncertainty was more in the background?
- Do you experience specific uncertainties when it comes to your work/hobbies and if so, can you describe them to me?

Follow-up questions
- Please describe the experience as if you were living through it again?
- Can you tell me what mood, feelings, or emotions arouse?
- Can you elaborate on that a bit further, as concretely as possible?
- What do you mean by…?
- What is it like for you to…?
- In what way…?

Final question: if you could describe uncertainty as a metaphor or image, what would it be?

3. Closing remarks

- Next appointment: observation in hospital.
- Verify contact information.
- Word of thanks and stressing of possibility to contact researcher at any time.

References

Dahlberg K, Dahlberg H, Nyström M. Reflective lifeworld research. 2nd ed. Lund (SE): Studentlitteratur; 2008. ISBN: 9789144049250.

Finlay L. Phenomenology for therapists: researching the lived world. 1st ed. Chichester (GB): Wiley-Blackwell; 2011. ISBN: 9780470666456.

van Manen M. Phenomenology of practice. Meaning-giving methods in phenomenological research and writing. 1st ed. Walnut Creek (CA): Left Coast Press; 2014. ISBN: 9781611329445.

van Wijngaarden E, Leget C, Goossensen A. Ready to give up on life: the lived experience of elderly people who feel life is completed and no longer worth living. Soc Sci Med. 2015;138:257-264. doi: 10.1016/j.socscimed.2015.05.015.

Weiss RS. Learning from strangers. The art and method of qualitative interview studies. 1st ed. New York: The Free Press; 1995.
